# Using Qualitative Research in Community Engagement to Capture and Mitigate Vulnerability Mechanisms in the Face of Infectious Diseases: Insights From a Research-Based Program in Five European Countries During the COVID-19 Pandemic

**DOI:** 10.1177/10497323251346407

**Published:** 2025-06-16

**Authors:** Mandy Geise, Jacob Osborne, Paul Grohma, Tamara Giles-Vernick, Benedetta Lana, Papa Mamadou Diagne, Zeliha Öcek, Uršula Lipovec Čebron, Neza Vodopivec, Anja Brunec, Jean Paul Baldacchino, Maurice Said, Gisella Orsini, Victoria Sultana, Concetta Vaccaro, Anna-Maria Volkmann, Ruth Kutalek, Michel Dückers

**Affiliations:** 1 Netherlands Institute for Health Services Research, Utrecht, The Netherlands; 2 Faculty of Behavioural and Social Sciences, University of Groningen, Groningen, The Netherlands; 3 Center for Public Health, Department of Social- and Preventive Medicine, 27271 Medical University of Vienna, Vienna, Austria; 4 Anthropology & Ecology of Disease Emergence Unit, 27058 Institut Pasteur*/*Université Paris Cité, Paris, France; 5 Pettenkofer School of Public Health, Munich, Germany; 6 197678 Rachel Carson Centre for Environment and Society, LMU Munich, Munchen, Germany; 7 Chair for Public Health and Health Services Research, Medical Faculty, 210417 Institute for Medical Information Processing, Biometry, and Epidemiology (IBE), LMU Munich, Munich, Germany; 8 University of Ljubljana, Ljubljana, Slovenia; 9 37563 University of Malta, Msida, Malta; 10 18388 Censis, Roma, Italy; 11 4919 University College London, London, UK; 12 ARQ National Psychotrauma Centre, Diemen, The Netherlands

**Keywords:** community engagement, vulnerabilities, social science research impact, infectious diseases, COVID-19, gender inequality

## Abstract

Various frameworks have been proposed for carrying out community engagement (CE) in the context of infectious disease response, but few have done so through a lens of vulnerability and even fewer, if any, have compared cases across countries. This paper reflects on the implementation of a project based on social science research and CE to capture and mitigate vulnerability mechanisms, which was carried out in France, Germany, Italy, Malta, and Slovenia during the COVID-19 pandemic. Using qualitative data collected through interviews, a focus group discussion, and project meetings, we describe how a stepwise CE process was carried out in the context of an international, multi-sectoral project. As such, this paper sheds light on the applicability of the methodology, the strategies followed, and overlapping themes encountered during the CE implementation. In all five countries, researchers created overviews of multiple vulnerability case descriptions situated in communities affected by the COVID-19 pandemic. Several recurring themes played a role in the CE process in the different settings: the challenges of finding, defining, and working with(in) communities; the role and position of researchers “in action”; stakeholders and power dynamics; timing of stakeholder involvement; translating qualitative data on vulnerability mechanisms into practical solutions; and sustainability and institutional integration. It is important to consider these themes when planning future initiatives to apply social and behavioral science methods to address and mitigate vulnerabilities in communities confronted with pandemics or other crisis contexts.

## Introduction

COVID-19 has had a devastating impact the world over, particularly in communities simultaneously contending with this infectious disease and various health, social, and economic disadvantages. These foregoing vulnerabilities severely affected morbidity and mortality from COVID-19 and were exacerbated by measures implemented to contain its spread (Da Mosto et al., 2021; Flor et al., 2022; Hayward et al., 2021; Laster Pirtle, 2020; Lipovec Čebron et al., 2024; Öcek et al., 2023). Prior epidemics have similarly revealed that marginalized populations experience higher mortality, increased morbidity, and more serious economic fallout (Fortuna et al., 2020; Singer & Rylko-Bauer, 2021). While marginalized people may encounter substantial barriers to accessing care and support, precisely how infectious diseases, existing vulnerabilities, and pandemic control interventions aggravate health and social vulnerabilities remains poorly understood. Insight into mechanisms of vulnerabilization (Zarowsky et al., 2013) in pandemic contexts can be crucial for developing multi-sectoral efforts to enhance their capacities to cope with current and future shocks.

This study is part of the EU-funded consortium Sonar-Global, which was established to create a global network of social scientists and to encourage the use of social science concepts and methods in the preparation for and response to infectious diseases and antimicrobial resistance (Giles-Vernick et al., 2019). Part of this work examined the impacts of the pandemic on populations facing health, social, and economic vulnerabilities and proposed concrete actions to mitigate the factors rendering them vulnerable. In five European countries—France, Germany, Italy, Malta, and Slovenia—local research teams implemented a framework for action (hereafter referred to as the action framework) through which they assessed mechanisms underlying vulnerabilities and explored together with community stakeholders and government actors how these vulnerabilities could be addressed in the short and long term. Using qualitative data collected during the implementation of the action framework, this paper reflects on the process through which Sonar-Global researchers in each of the five countries used this process to operationalize context-specific qualitative insights on COVID-19 for community-centered action and policy. Here, we provide deeper insight on community engagement by describing the process from a multi-country, multiple case study of an approach rooted in social science methodologies. In doing so, we address an important gap in the literature on social science in (and of) community engagement.

### Sonar-Global’s Research Structure

**Figure 1.**
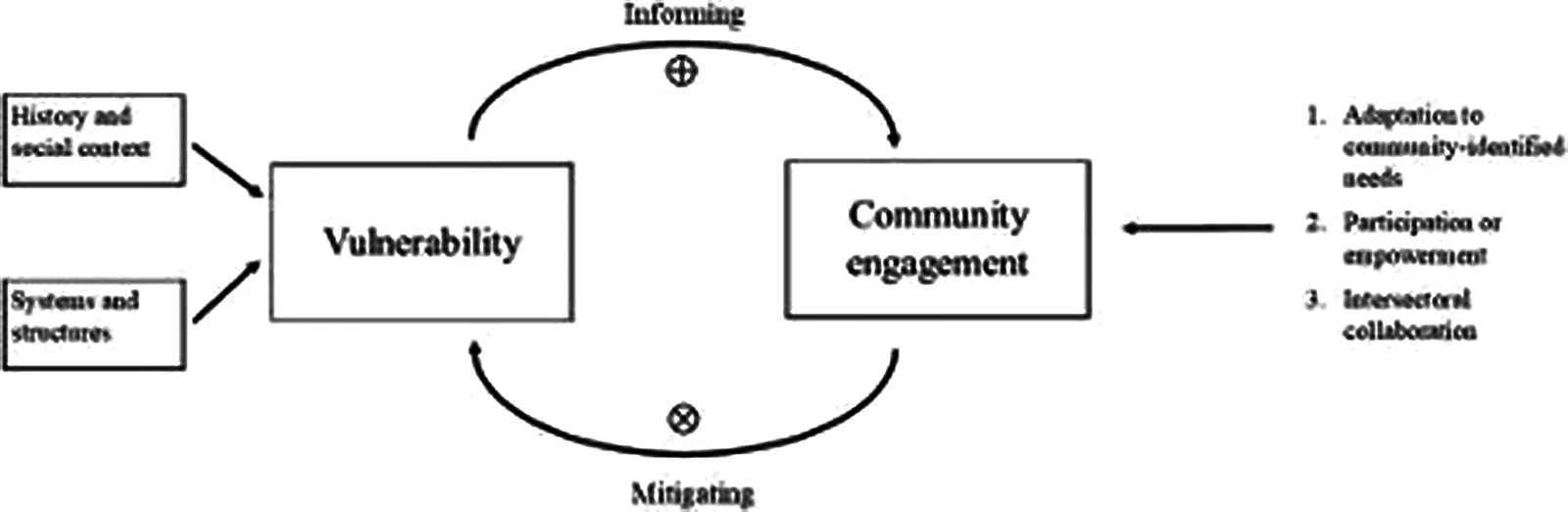
The cyclic relationship between vulnerability and community engagement (Osborne, Paget, Giles-Vernick et al., 2021).

The project’s approach was based on an action framework containing two main elements: a Vulnerability Assessment (VA) and Community Engagement (CE). The VA is a qualitative tool to assess vulnerabilities through context-adaptable methods, which has been modified from its original application in humanitarian crises for use in the context of COVID-19 (Napier, 2013; Volkmann et al., 2021). Drawing on qualitative insights from the VA, research teams engaged community stakeholders in the five project countries and collaborated with them to provide actionable solutions to the identified vulnerability mechanisms to local stakeholders, policy makers, and academic communities. In this way, the data collected in the VA phase informed the CE activities, allowing for adaptation to community-identified needs, participation of community actors that could have otherwise been missed, and intersectoral collaboration. Through working with community stakeholders to take stock of findings and formulate recommendations for what could be done to address context-specific mechanisms that make people vulnerable, CE can mitigate vulnerabilities in different areas (see Figure 1), in the short, medium, and long term. The VA–CE action framework that is based on this cyclic mechanism is informed by social science literature on both topics and described in Osborne, Paget, Napier et al. (2021). The concepts of vulnerability and community engagement are framed by social science literature as well as biomedical research and epidemiology in relation to infectious disease science (Osborne, Paget, Giles-Vernick et al., 2021).

Within Sonar-Global’s CE activities, stakeholders worked together to identify and develop action points and policy recommendations based on the insights from qualitative research in each country. In all cases, COVID-19 was used as the topic of focus that bound the activities in which social scientists worked simultaneously to understand vulnerabilities through qualitative research and work toward actionable solutions together with community stakeholders. Similar approaches have been used in other infectious disease contexts, including Barker et al. (2020) in the case of Ebola virus disease, Tangseefa et al. (2019) for malaria, and Burns et al. (2020) and Hussen et al. (2018) for HIV. In this paper, we focus on the project’s CE activities, during which the research teams collaborated with stakeholders to identify and develop action points. The VA activities that took place in the first phase are described in the respective country reports shared on the Sonar-Global website as well as peer-reviewed papers on the case studies in Germany (Basili et al., 2025; Öcek et al., 2023), Italy (Vaccaro et al., 2023), and Slovenia (Lipovec Čebron et al., 2021, 2024).

### Key Concepts and Theoretical Framing

Sonar-Global’s approach to understand situated vulnerability and resilience mechanisms relied on CE as a participatory process to ensure action and policy could be used for application in a relevant and meaningful way. With different contexts, the exact CE content and shape differed between the countries but was always embedded in the same understanding of engagement. While there are numerous interpretations of the format, role of communities, and operationalization of CE, this project followed the definition of CE described in the UNICEF Minimum Quality Standards for CE:

A foundational action for working with traditional, community, civil society, government, and opinion groups and leaders; and expanding collective or group roles in addressing the issues that affect their lives. Community engagement empowers social groups and social networks, builds upon local strengths and capacities, and improves local participation, ownership, adaptation and communication. (UNICEF, 2020, p. 6)

In this understanding, CE thus aims to directly involve communities in dialogue, decision-making, implementation, and policy on issues that affect them, shape their access and use of services, improve their well-being, and strengthen resilience. The latter is a concept that has been used to signify a counter to vulnerability (Perez-Brummer et al., 2017). Building on a participatory approach and existing capacities, CE aims to strengthen community capacities as well as community networks and ownership so that communities can lead on issues that affect them (UNICEF, 2020). This does not mean placing the burden and responsibility of fostering resilience or empowerment on the shoulders of communities, which Popay et al. have signalled as a worrying trend in public health or health promotion initiatives, as state services “shrink and social and health inequities widen” (2021, p. 1253). Rather, we propose CE that takes into consideration power imbalances, structures, and mechanisms that can leave (some) community members vulnerable by signalling those very mechanisms through qualitative research in the VA and then address those in collaboration with communities, facilitating the inclusion of their experiences and needs. This form of CE seeks to support the development of capabilities not only through individual capacities and proximal neighborhood conditions and access to services—considering community resilience with what Popay et al. (2021) see as an “inward gaze”—but also seeks to have greater resilience embedded through institutional changes, wielding an “outward gaze” on sustainable political and social transformation. It is also attentive to the complex power dynamics at play between different community stakeholders and integrates this understanding into its design and implementation.

Within the action framework, researchers engaged with local community stakeholders including national and local government actors, civil society organizations working with vulnerable groups, and affected communities in dialogues to discuss findings on vulnerabilities and work together toward the formulation and/or implementation of actions and recommendations. The conception of community remained flexible throughout the process. CE was approached as a means to identify, understand, and respond to community needs, and a way to build community, as well as a mode of citizen engagement that can aid in ameliorating the negative effects of vulnerability. One of CE’s well-known predicaments is the issue of using CE in a participatory and inclusive way (Johnston & Lane, 2021). CE is sometimes used as tick-box exercise and often hampered by assumptions that the community is a homogenous unit, by people being (inadvertently) left out, and by the more powerful or vocal stakeholders dominating or even hijacking the process (Johnston & Lane, 2021). Successful participatory processes require openness of dialogue with a genuine empathy for others’ perspectives; active listening; early and ongoing involvement; and creating meaningful decision-space throughout the engagement process (Heyerdahl et al., 2021; Johnston & Lane, 2021; UNICEF, 2020).

Vulnerability has been described in many contexts and through several conceptual framings. Within the context of COVID-19, we have seen vulnerability used to refer to individual risk of severe infection (DeCaprio et al., 2020), to the effects of policy-shaping in pandemic responses (The Lancet, 2020), and to systemic and structural vulnerabilities that emerged from existing social dynamics (Cantos & Rebolledo, 2021). The Sonar-Global action framework focused on vulnerabilities provoked or exacerbated by COVID-19, while recognizing that many of the factors that make people vulnerable existed long before the pandemic. Here, we consider vulnerability to be the consequence of interacting, dynamic factors that can propel people into or out of conditions of marginalization or exclusion which result in harm to health and well-being (Linder et al., 2018; Luna, 2009). We are particularly focused on the historical and social contexts of locally identified structures within which marginalizing mechanisms operate (Osborne, Paget, Giles-Vernick et al., 2021).

Within the context of community organizing and enacting transformative change, connections to more political forms of vulnerability come to the fore, including Stephenson et al.’s (2014) description of vulnerability and its relation to public health governance in pandemics and Zarowsky et al.’s (2013) emphasis on how policies should respond to the dynamics of vulnerability. These concepts, together with the above description of the literature grounding engaging with communities through a deep understanding of their context, are what form the basis of how we understand the reflection of the CE process used within the Sonar-Global project. This paper does not intend to quantify impact but reflects on how the different research teams applied strategies in which they gathered and adapted qualitative insights about situated vulnerabilities for community-centered action in participatory processes. It considers the ways in which each country case’s process involved community stakeholders in dialogue, formulation, and implementation of the action recommendations, the instruments used for the collaboration, and the potential sustainability. A further synthesis of this framing and its application for other contexts is taken up in the Discussion section.

## Methods

Based on a systematic literature review on vulnerabilities and CE and following the principles of the UNICEF Minimum Standards for CE (2020), the CE coordinating team drafted an action framework that was subsequently shared with the five country research teams and finalized with their input. The CE coordinating team gave individual and group support to the country teams as they went through the action framework’s steps to integrate the qualitative research findings into the development of CE activities on a country level, such as the organization of community events to present and receive feedback on their findings, and formulate action recommendations.

As the CE activities progressed, the coordinating and country teams reflected on the process of using qualitative research in CE, applied in the context of vulnerabilities engendered or exacerbated by COVID-19. The aim was to document and unpack how this process unfolded in five different settings and to identify and analyze facilitating factors and barriers formulating and implementing solutions with community stakeholders, from the perspective of social scientist researchers. This paper provides both insights into the design and implementation of the CE activities as well as the strengths and challenges of the situated processes. Throughout the process, which lasted from July 2021 to July 2022, the coordinating team observed CE activities organized by the country teams, such as stakeholder meetings, and collected data in bi-weekly meetings where country teams gathered to discuss their approach, progress, and challenges. Toward the end of the project, the coordinating team conducted seven in-depth interviews with members of the different country teams and organized a focus group discussion (FGD) with fourteen participants. The bi-weekly meetings, interviews, and FGD were conducted online due to the geographical spread of the coordinating team and country teams, and under mobility restrictions related to COVID-19, to allow all of the researchers involved in the CE activities to participate and share their perspective. As all teams had been meeting with each other through video calls regularly for over a year when the interviews and FGD started, all participants had become well-accustomed and comfortable with meeting with each other online.

The semi-structured interviews were conducted by the CE coordinating team with the local research teams that conducted the VA and CE activities in each of the five project countries. The coordinating team conducted interviews with the country team members after they had carried out their second or third round of dialogues with stakeholders, from the end of 2021 until mid-2022. The interviews were meant to get an in-depth understanding of how country teams had experienced certain aspects of the process—engaging with stakeholders, navigating power dynamics, making the qualitative data from the VA accessible for providing insights to different community stakeholders, co-designing actionable solutions from the findings, ownership, and sustainability. The FGD was meant to obtain a comprehensive understanding of how the process was experienced by the different researchers in the project through collective discussion. The FGD, in which two or three members from each country team participated, was similarly used to reflect on the CE and how the VA informed this process, such as the selection of stakeholders based on findings on vulnerability mechanisms. The coordinating team asked country team members in the five countries specifically about the process of involving stakeholders, communicating the findings on mechanisms that can make people vulnerable into clear points to present to their interlocutors when engaging with different community actors, and the challenges and opportunities encountered.

Additionally, the researchers from the coordinating team followed and supported the VA–CE trajectory through bi-weekly collective meetings with all local research teams as well as through regular individual meetings with the teams in which strategy, organization, target groups, and format of the CE protocol and meetings with stakeholders were discussed. Another set of data came from unstructured participant observation of CE events with community stakeholders in the different countries and from the materials and discussion notes stemming from those events. Coordinating team members were present either in person or online, sitting in on the discussions about the findings on vulnerability mechanisms and co-creative processes on the formulation of action points and recommendations. This involved observing plenary presentations and reflections, smaller break-out groups, and work sessions with different community stakeholders and facilitated by the country teams. To ensure our findings regarding the CE process were comprehensive and to avoid blind spots as we developed and piloted the action framework, we opted for observation to capture all aspects and issues that might arise during these meetings and to trace the dynamics of the different formats, which are described in further detail in the Implementation of the VA–CE Framework section.

The interviews and FGD were recorded and the recordings were transcribed and pseudonymized. Notes that had been taken of the CE events in the individual countries and during the bi-weekly collective meetings with the research teams were pseudonymized. English was spoken during the interviews, the FGD, and the bi-weekly reflection meetings. Participant observation of stakeholder meetings was done by team members proficient in the language spoken during the event, and observation notes were taken in English. The transcripts and notes were then coded in the qualitative data analysis software Dedoose by two researchers according to consensus coding, and a coding tree was developed based on thematic analysis. The thematic analysis inductively identified several themes that describe the aspects the project’s researchers experienced when doing CE within the action framework. Lessons were drawn about how researchers can put qualitative data to use for participatory and inclusive work to address vulnerability mechanisms with community stakeholders in the context of infectious diseases. The identified themes, strengths, and challenges provide insights into what approaches can help to make sure that proposed solutions and recommendations will reach the right desks and minds to make an impact.

## Results

The results are divided in two subsections. First, we present results on the implementation of the action framework in each study setting. We then detail the themes that characterize the process, considering facilitating and challenging factors, the types of stakeholders that research teams involved to ensure development and uptake of co-created solutions, and ways to facilitate sustainable collaborations. To focus on the characteristics of the process and to protect the researchers and community participants in politically sensitive contexts, we will not use the specific country names but refer to the five case study countries as Countries 1, 2, 3, 4, and 5. Quotes and other references to data are notated with the method type: FGD, interview, stakeholder event, and partner meeting.

### Implementation of the VA–CE Framework

The action framework’s stepwise approach was tailored to the context of each of the countries, so that each research team presented findings on situated vulnerabilities to local stakeholders and sought actionable solutions that best fit local needs, social, and political context. The different local strategies country teams employed within the action framework are described below.

#### Country 1: Local Advisory Committee and Direct Participation

The research team in Country 1 engaged with stakeholders early in the process. As its researchers were collecting the data for the VA, they also organized meetings with local and national NGOs, local government officials, and healthcare providers. These stakeholders made up a Local Advisory and Action Committee, with whom the researchers could engage in ongoing presentation and discussion of the findings. The constitution of this Committee was developed by conducting informal interviews with stakeholders prior to the conduct of the VA. Local government was interested in active collaboration with the research team and in exploring possibilities to use the VA insights for further research and local policy making, together with other partners (FGD, 03-12-2021). Direct participation in partnerships and multi-stakeholder efforts against exclusion and marginalization meant a “concrete invitation to participate in an ongoing dynamic” (FGD, 03-12-2021). In seeking a link to ongoing activities and dynamics and nurturing efforts to address mechanisms underlying vulnerability with their VA data, the research team in Country 1 effectively could build upon structures and initiatives already happening. The team considered that be(com)ing embedded in long-term and/or existing institutionalized structures heightened the chances of sustainability of their work (FGD, 03-12-2021).

#### Country 2: Key Community Representatives for Strategic Engagement

The research team in Country 2 organized additional interviews after the VA as part of an approach to engage more community-based actors in the process of formulating action recommendations informed by the findings on vulnerability mechanisms. By talking to actors with key positions in connection to vulnerable people, such as women’s health practitioners, members of local NGOs and of migrant, cultural, and religious associations, and local government representatives, the team acquired additional insights into vulnerability mechanisms and amplified opportunities to work with community stakeholders on the formulation of recommendations and further collaboration. This resulted in a stepwise approach in which diverse community stakeholders provided feedback on the initial findings from the VA and the additional understandings of vulnerability mechanisms, as well as ways to address them that emerged in the multiple rounds of discussion. In three reiterative rounds, consisting of an online open forum and multiple in-person events, this research team received feedback from community stakeholders on the VA findings and the actionable solutions the research team had proposed in different domains, including healthcare and COVID-19 prevention, social support, and human rights, living, and the housing environment. The process included several adaptations of the action recommendations to account for input from different stakeholders reflecting their experiences, perspectives, and needs. Overall, stakeholders included key community people and experts (healthcare professionals, cultural leaders, people with disabilities, and migrants), local government, and public health agency officials. The subsequent events were also considered instrumental in reaching more community stakeholders, and in building ongoing relations through a common goal or objective to formulate and reformulate the action recommendations, increasing commitment from stakeholders to make sure the findings and recommendations “reached the right ears, eyes and desks” (stakeholder event, April 2022). Building long-term relations with key community stakeholders and local policy makers was seen as an opportunity to obtain a sustainable result (interview, 31-05-2022).

#### Country 3: From Community Stakeholder to Policy Makers

The research team in Country 3 organized two CE events on different levels, one with stakeholders working with vulnerable populations and one with policy makers. The team aimed to mobilize regional and local governmental actors in implementing some of the policy recommendations, to bridge the divide between national policy and local action (interview, 07-06-2022). The team was able to do so because of long-standing collaborations with policy makers, healthcare professionals, and NGOs on a national and local level. A first meeting was organized in March 2022 with stakeholders involved in pandemic activities at different levels: GPs, medical specialists, including psychiatrists, teachers, psychologists, social workers such as representatives of Catholic voluntary organizations, members of patient advocacy organizations, healthcare professionals and social workers, and university professors. In the research team 3’s stepwise process, the first event served to present and consult on the findings of the VA and collect input on the recommendations. The second event served to translate the recommendations into policy formulation and setting with policy makers. One of the key actionable policy recommendations to address compounded vulnerability mechanisms that the research team is currently rolling out is the training and installment of ‘Health City Managers,’ who take an integral approach to health and well-being. A program to train these managers has currently been piloted in ten cities (interview, 07-06-2022).

#### Country 4: Coalition of Goodwill

The research team in Country 4 consisted of academic researchers and included a researcher affiliated with the national mental health services. The team engaged stakeholders including (mental) healthcare workers, representatives from government agencies, and local NGOs. At the beginning of the VA, this team entered into an agreement with several organizations working with vulnerable people, by signing a Memorandum of Understanding (MoU). The MoU contributed to a sense of commitment among partners and was beneficial to communicating on vulnerabilities and solutions, as well as reaching certain vulnerable groups, with some members functioning as liaison. The CE event the research team organized had many participants from organizations that were part of the MoU, which, through ongoing exchange of findings as part of the study, had fostered a sustainable “coalition of goodwill” (FGD, 03-12-2021). Stakeholders could discuss their perspectives on factors that contribute to vulnerability and resilience in the context of the COVID-19 pandemic. These were used to draw additional insights regarding vulnerabilities, feeding back into the analysis loop. The stakeholder dialogue furthermore included break-out discussions on policy recommendations.

#### Country 5: Diversified Focus to Strengthen Sustainable Collaboration of Community Stakeholders in Urban and Rural Settings

The research team in Country 5 have a long history of working with some of the communities and respective stakeholders they reached out to for the VA and CE activities. The researchers could build upon that network to expand and reach an even more extensive and diverse set of stakeholders in multiple settings. Stakeholder groups were targeted and connected around topics, geographic location, and institutional affiliation. Partially because they experienced political unwillingness or even hostility, the research team in Country 5 focused on local actors to effectuate change. The team organized different events in the country capital and the remote rural region where they worked to balance interests and manage tensions between different stakeholders. In these events, stakeholders discussed vulnerabilities according to eight realms the researchers had identified in the VA, adapted the action recommendations drafted by the research team, and proposed additional recommendations. Collaboration was stimulated between stakeholders within the relevant realm. The team was careful to avoid a meeting that would get stranded in a generalist discussion as issues might feel too distant between stakeholders in the capital and in the rural region. Aiming to ensure an environment of trust for all stakeholders conducive to a productive discussion about the VA insights, the research team organized three stakeholder dialogue events. Organizing dedicated stakeholder events for the different locations aligned with the organization and findings of the VA, and also would make it possible to address the specific issues present in the two localities. Bringing the different viewpoints together was seen as a next step. Based on the vulnerability analysis and stakeholder events, the research team issued a set of action recommendations. The team sought additional dissemination and dialogue opportunities with researchers, media, and public health experts.

As can be seen in Table 1, similarities across the different country approaches include a collaboration where social scientists lead the VA–CE process, data collected is based on VA interviews, and stakeholder events are organized in which VA findings are validated and policy recommendations formulated. Unique elements in the approaches that aid the CE process and its sustainability include building upon existing structures, networks, and initiatives (all Country Teams), local advisory committee (Country Team 1) and experts (Country Teams 2, 4, and 5), initiating a formal coalition through a memorandum of understanding (Country Team 4), and an online forum (Country Team 2).

**Table 1.** Features of CE in the Different Countries—Showing Similarities and Differences.

Country Team	1	2	3	4	5
Social scientists in lead^a^	+	+	+	+	+
Data collected based on VA interviews	+	+	+	+	+
Organized meetings to validate findings	+	+	+	+	+
Policy or action recommendations formulated	+	+	+	+	+
Building long-term relations	+	+	+	+	+
Additional interviews before or after VA to include more (different) community stakeholders	+	+			
Collaborate with service providers: Care and social support, NGOs	+	+	+	+	+
Work with local government actors	+	+	+	+	
Embraced by local government	+		+		
Collaboration with national government actors			+		
Built upon existing structures and initiatives	+	+	+		
Building upon existing long-term engagement with vulnerable people					+
Advisory Committee of stakeholders	+				
Online open forum		+			
Scaling up			+		
Memorandum of understanding				+	
Media output about findings and activities				+	+

^a^Country Team 2 began with a social scientist leading the VA–CE activities but was later led by a public health scientist.

### Thematic Analysis of the VA–CE Framework Process

In this subsection of the results, we further examine the process of applying the VA–CE action framework based on a thematic analysis of the research teams’ experiences as they established different modes of engagement with communities and the co-creation of solutions. From our analysis, several transversal themes emerged that exemplify key issues marking social science–based CE efforts based on infectious disease contexts. The themes are elaborated upon below. They include (1) finding, defining, and working with communities, (2) timing of stakeholder involvement, (3) translating qualitative data on vulnerability mechanisms into practical solutions, and (4) sustainability and institutional integration.

#### Theme 1: Finding, Defining, and Working With(in) Communities

As the different country cases show, communities and vulnerability mechanisms are fluid. The healthcare professionals, local government officials, social workers, NGOs, and researchers working with people that are vulnerable were also diverse, and their positionality and ability to influence the mechanisms that can make people vulnerable on a micro or macro level were inconsistent.

The local team researchers experienced challenges in defining the community and its related stakeholders as an object of study and intervention. This was especially challenging with the aim to remain open to putative new—“unknown”—vulnerability mechanisms and finding people in the community that often are invisible. Even if the VA helped to identify stakeholders, local research teams encountered challenges in finding and defining communities without preconceived notions:

We worked in places where pre-supposing what a community is, it’s been so problematic. We wanted to go into the project without dogmatic notions of what community is, wanted it to emerge before us. But that can make it harder to draw a line and define who falls within and who outside, since there are many different forms of vulnerabilities. (Country 1 researcher (FGD, 03-12-2021))

Local research teams had to remain attentive to new vulnerabilities and communities that might present themselves in unexpected forms. This involved an ongoing (re)defining and (re)demarcating of how they understood the communities and vulnerabilities they worked with and resolving how to best find and involve actors who face vulnerabilities, but also a process of figuring out how to reconcile input from community actors with different social and political capital and varied levels of interest in collaborating. All local research teams had some initial ideas of populations who might be more vulnerable in the context of COVID-19, which were concentrated in communities most affected by structural inequalities. As the VA and CE activities progressed, understandings of who might be affected by (what kind of) vulnerability mechanisms shifted, and different stakeholders were included. As a researcher from the team in Country 5 put it, “with the VA we needed to look for invisible vulnerabilities and be open to newly emerging vulnerabilities” (interview, 07-06-2022).

In some situations, researchers complemented the VA interviews with additional interviews, for example, to get a better sense of how vulnerabilities were affecting people’s lives and who was affected, ensuring not to miss people who might not fall within the categorical notion of vulnerability. To begin recruiting participants, the researchers in Country 1 started to engage with NGOs working with marginalized people that could help them to reach out to those populations and at the same time function as key experts on vulnerability mechanisms in affected populations. Researchers also conducted additional interviews to reach different kinds of community members and to get more (diverse) community members involved with the formulation of action recommendations. The team in Country 2 interviewed over 100 participants in the VA, but the first CE events they planned received such a low number of confirmations for participation that they decided to cancel the events and re-strategize. The team then expanded the base of stakeholders to collaborate with, by conducting interviews with 20 key community people and experts, who helped them gain access to the community.

##### Sub-Theme 1.1: Stakeholders and Power Dynamics

Each research team presented findings on situated vulnerabilities to local stakeholders and sought actionable solutions to these that were captured in policy or action recommendations, according to the vulnerability factors they identified in their local setting and with the stakeholders they perceived as valuable and strategically effective to engage with. Teams selected stakeholders based on an analysis of the local stakeholder spectrum, which included public administration, health and social institutions, as well as NGOs and individuals representing socially, politically, or economically vulnerable populations affected by COVID-19 or working in pandemic response. Initial contacts with key stakeholders were extended through a snowball method (Baldacchino et al., 2021; Giles-Vernick et al., 2021; Lipovec Čebron, 2021; Öcek et al., 2023; Vaccaro et al., 2023). Stakeholders included government actors (e.g., Ministry of Health, national public health institute, or municipality), (mental) healthcare professionals, social workers, organizations working with migrants, the older adults, people without access to (healthy) food, and cultural or religious associations; but also other researchers seeking to exchange complementary data and develop a more comprehensive understanding of the factors underlying vulnerabilities. As heterogeneous groups, these stakeholders had differing objectives, perspectives, influence to enact (some of) the action recommendations, and proximity to the community. In relation to representability and including community voices in community efforts, Country 5 researchers indicated having to go through “layers” to get to the most vulnerable: “First you get to the people who are willing to speak. These are not the most vulnerable. But then you go on and reach more people” (FGD, 03-12-2021).

A team member from Country 1 underlined the heterogeneity of community stakeholders, most notably between the people facing vulnerabilities—in this case beneficiaries of social support—and the stakeholders working with them:

For instance, people who were working with asylum seekers or people who were working with beneficiaries at a food bank were not in the same situation as the beneficiaries, nor did the recipients see the people working in those NGOs as in the same group as they were, or in some kind of community. (FGD, 03-12-2021)

In their ongoing engagement with stakeholders through the Advisory and Action Committee, the researchers from Country 1 maneuvered challenging dynamics between stakeholders who came to the table with varying degrees of collaborative and policy experience but who had not previously worked together. There were tensions over clashing interests and initially some stakeholders dominated the discussion. The team said that they were able to temper this through moderating the discussion and ensuring everyone had a chance to speak.

The team from Country 5 carried out their work in the capital and in a rural border region. Based on the differences in the complex interplay of factors making people vulnerable and the disparate stakeholders in the capital and rural region, the team decided to engage with different stakeholders in separate events. The team believed a smaller, focused group would have most impact. Having dedicated events in the capital and the rural area would not only ensure stakeholders were able to talk more pointedly about mechanisms underlying vulnerabilities most relevant for their area and communities but also that stakeholders would engage more directly with collaborating parties in working toward solutions. It would also avoid antagonism, which the researchers had identified as a possible barrier to fruitful discussion with stakeholders with diverse, at times incompatible, interests at the table, as well as possible resistance from certain stakeholders to engage in “policy talk” or an institution-centric approach, political tensions between stakeholders, and a lack of interest from national-level policy makers to talk to grassroots organizations (project meeting, 02-09-2021).

Researchers indicated that discussion between stakeholders at times relied on certain power dynamics to encourage collective action; if one party was on board, then other stakeholders might be more inclined to join, too. For the researchers from Country 4, the stakeholder event was appreciated by participants as an opportunity to connect with others working on the same issues, and the chance to “come together and learn from each other on what’s being done in the country and how we can build on this” (stakeholder meeting, 31-11-2021). It established common ground for discussion and discovery between those open to exploring joint efforts, such as a centralized platform through which citizens could access social, health, and financial support in one place rather than having to navigate the fragmented support landscape.

##### Sub-Theme 1.2: The Role and Position of Researchers “in Action”

As researchers engaged with stakeholders to use their findings on vulnerability mechanisms to formulate action points to address these, they navigated a landscape where roles, dynamics, and issues at stake were not always clear-cut. This included their own roles. All project researchers were convinced that community stakeholders needed to be at the center, but they were constantly working out their own position in relation to the community stakeholders, the findings, and the impact it might have.

Several of the project researchers did not consider themselves as having the resources—network and capacities—for a role of advocacy. A member from the team in Country 2 said, “We are far from this, we don’t have the infrastructure, we are only some researchers” (FGD, 03-12-2021). This researcher lamented the limited influence of research findings and action efforts if the research team is not embedded in a network of local, influential institutional actors. However, the teams in Countries 1, 2, and 5 showed that with persistence, sustained effort, and finding the right key persons, it is feasible—and the stepwise approach is helpful—to reach community stakeholders and access such networks and build further on long-term collaborations. Country Team 3, which already had an extensive network and working relationships with national stakeholders in ministries and parliaments, made suggestions for policies regarding education and availability of resources for exercise, green space, and play, which were incorporated into new laws (interview, 09-06-2022).

When shifting from research to action, researchers sometimes felt out of their depth in the action role, especially when it was about steering and balancing communities’ interests and finding common ground with policy makers without weaponizing and polarizing and keeping all parties interested. A researcher from the team in Country 1 mentioned they would have liked to have some guidance from the beginning of the project on how to work with stakeholders, to encourage stakeholders to be open to new perspectives and issues and also to bring them back into the fold in the case of fatigue. The team researchers felt it would be beneficial to be better prepared for managing stakeholder expectations and influence, and to have the skills to act as a negotiator (FGD, 03-12-2021).

However, there was also consensus that researchers can effectively support communities and their needs in becoming part of policy making processes. Project researchers felt that social scientists are uniquely equipped to provide evidence in collaborative and co-creative efforts with communities through their attention to positionality and relationality, encouraging open participation. For CE to be truly effective and attentive to the needs of communities, as Country Team 4 member mentioned, “you have to build all interventions around the reality of people” (FGD, 03-12-2021).

#### Theme 2: Timing of Stakeholder Involvement

Country research teams all agreed that stakeholder involvement should occur as early as possible in the process (FGD, 03-12-2021). This can offer community actors the chance to be involved in research design, formulate relevant questions and objectives that fit their needs, and also allow time to build trust and relationships. In the project’s design, early CE was not planned, and this was experienced by all the local research teams as a weakness.

However, some of the research teams did engage with community stakeholders early in the process. Researchers in Country 1 brought in their stakeholders while the VA was still ongoing. The researchers in Country 3, making use of existing relations with local and national policy makers as well as NGOs and healthcare professionals, had a chance to collect insights from these stakeholders while they were conducting the VA. In these cases, the research teams found that such early involvement contributed to more clearly formulated vulnerability mechanisms and courses for action to address these. This benefits a more involved and inclusive process, and as such involving stakeholders from the beginning of the project is a recommendation.

#### Theme 3: Translating Qualitative Data on Vulnerability Mechanisms Into Practical Solutions

The process of formulating and reformulating recommendations included several adaptations of the action recommendations to account for input from different stakeholders reflecting their experiences, perspectives, and needs. As the teams looked at common points and connections, the recommendations were divided into general or transversal recommendations and those that were more applicable to, and formulated together with, specific groups such as migrants, refugees, people with mental health problems, older people, and healthcare workers.

Recommendations addressed public health measures that severely impacted the social, economic, and health resilience of vulnerable people, but also to invest in broad social and societal prevention. For the research team in Country 3, which worked closely together with national and local policy makers, the VA–CE action framework was built around the strategy to gather experiences and perspectives “on the ground” from vulnerable people and those working with them, and then take these “up” to (national level) policy makers (interview, 07-06-2022). The recommendations formulated by the research teams in Countries 4 and 5 were aimed at transversal efforts. They were not given per group, but instead geared toward mechanisms that can mean better outcomes and resilience for many different people facing vulnerabilities.

Project researchers felt they were well equipped in terms of understanding the issues and the data they had collected. The richness of the data and the chances at sustainable engagement were experienced as a positive aspect of making the qualitative data applicable for action and policy making. People’s lived experiences were brought to the level of evidence by synthesizing findings on what made people vulnerable and organize them into categories or domains. The research teams used these data, which included descriptions of how people were affected by vulnerability mechanisms. In stakeholder dialogue meetings, discussion and several adaptations of the action recommendations made sure different stakeholders saw their experiences and perspectives incorporated, and action was designed to match these.

#### Theme 4: Sustainability and Institutional Integration

The research teams agreed that CE events in the project served sustainability in two ways: by bringing actors with shared interests to the table, establishing common ground for discussion and exploring intersecting issues; and by presenting and garnering ideas on how solutions to these issues should be jointly formulated and implemented. Researchers were hopeful that the Sonar-Global activities started or cultivated ongoing collaborative efforts.

All partners have established a process to address COVID-19-related vulnerabilities through the VA–CE action framework, with the intention to have sustainable collaboration and partnerships, implementation of recommendations in policy, and the scaling-up of data. This is a first step in working together and showing the strength of social science insights and methodologies, including for collaborating with community actors, which is key to effective and inclusive policy making.

Three research teams focused their efforts on the local municipal level: The research team in Country 1 has set up ongoing working relations with local municipal policy makers as well as research initiatives. The Country 2 research team is working together with local council members working on issues affecting migrants and people with disabilities, and with the provincial public health institute. The team is continuing to collaborate with the network of migrants and key community people it had established, on further research and action. In Country 3, a key strategy to address vulnerabilities and promote more resilience among residents in the larger cities that the team is currently implementing is the training and installment of “Health City Managers,” who take an integral approach to health and well-being. The country team has been training Health City Managers in the country’s largest cities. The Country 4 research team has begun a collaboration with several local NGOs and health services. The research team in Country 5, whose researchers have a long history of working with some of the communities and respective stakeholders they reached out to for the VA and CE activities, strengthened and diversified their network, reaching an even more extensive and diverse set of stakeholders in multiple settings to collaborate with.

## Discussion

Reflecting on the CE process in the five countries, it was possible to organize a structured VA–CE process in multiple countries. The approach reflected local idiosyncrasies, while identifying recurring themes. Project researchers were careful to avoid tokenism and committed to long-lasting, structural commitment from institutional and community actors, and investment (funding) and collaboration beyond project-based and episodic efforts.

The last years have seen a growing commitment from international and national government (and non-government) actors to set up structures that acknowledge the importance of people and community-centered action (Nigeria Centre for Disease Control, n.d; WHO, 2017), also often with an eye for broader social factors (UNICEF, 2020). It is necessary to continue and bolster these efforts, and allow for long-term research and action that might take a longer time to take effect. Solutions are only likely if different stakeholders work jointly to define problems and create synergy in the community’s problem-solving capacity. It may be beneficial to place a community actor—or a team of actors—leading the effort, who understands the interdependent relations, the stakes, and the background of the issues. Stakeholders that have a close connection to the community (and a high interest in the implementation of proposed actions) are also essential to avoid a millefeuille of institutional layers between affected communities and policy makers.

### Strengths

Qualitative data methods provided important insights on experiences of vulnerability by different groups that otherwise would have been missed, a strength described in studies of CE elsewhere (Ashworth et al., 2021; Jeleff et al., 2022; Osborne, Paget, Giles-Vernick et al., 2021). While pre-selecting groups that fall within categorical definitions of vulnerability is a traditional methodological step, being open to discover new vulnerabilities was a good practice that helped project researchers learn new, unexpected aspects that might feed into vulnerability, and go outside of expected groups. Here, we may use Luna’s (2009) description of “layers” of vulnerability or MacGregor et al.’s (2022) “intersecting precarities” to illustrate the dynamic and context-driven nature of vulnerability. This helped to identify communities and stakeholders with whom to engage and how engagement could best be carried out. As such, the CE described here contributed to ownership of findings and input for solutions based on including the right people, talking about problems and solutions that were relevant to them, through an approach adapted to the social, economic, political, medical, and geographical landscape.

Future CE should likewise be organized to uncover, understand, and address vulnerabilities within and beyond infectious disease and even broader crisis contexts. Taking a broader view of how social, economic, and political factors can influence health and well-being across communities, the action framework would be a valuable contribution not only to further infectious disease and disaster research and action but also for public health promotion under “normal” circumstances (Adhikari et al., 2020). As in the present example, including such a framing makes clear how inequalities are embodied and situated within particular contexts.

### Challenges and Lessons to Draw From

Some of the challenges occurring in other projects that seek CE also appeared here. Engagement of community stakeholders and effectuating changes on a policy implementation level are long-term processes. Some of the partners could rely on existing networks, and some partners had to build starting these relationships from scratch. VA findings indeed informed solutions, but while collaboration on community-supported solutions was promising, progress on implementation and integrating into policy has been modest. As discussed in Themes 1 and 4, getting the right institutional actors to listen and act, especially for the long term, was a barrier in the research-to-policy translation. At times, researchers ran into the limits of what they and their partners could influence with the resources, time, and political and social power at their disposal. This also depended on the inclusion and will of institutional actors, especially policy makers; these can be a great barrier or support in turning research findings and recommendations into policy and action. Inclusion of topics and access of groups to the table proved an issue—actors with (decision) power can dominate the discussion and/or be a barrier to contributions from others. It could be challenging to engage some community stakeholders, not necessarily to reach them but to get them on board and participate, for a diversity of reasons: lack of trust or time, fear, anonymity, lack of interest or competing interests, tensions between stakeholders, and perceived relevance or benefit (FGD, 03-12-2021).

There are differences and complementarities between participation, engagement, and involvement; engagement does not guarantee participation or ownership (Brunton et al., 2017). Levels of participation were difficult to measure in the project, but it was not a main objective to do so; hence, there were no instruments designed to evaluate it. Eventually, to understand the impact of the actionable solutions and sustainability of collaboration with stakeholders in community-centered research, more is needed. For future projects, an evaluation of the situation beforehand would provide a baseline for comparison. There is a need for more empirical work to develop and apply explanatory theories, frameworks, and models to better understand how participation occurs, under what contextual settings, and what is produced.

The methods described within the Sonar-Global research activities may serve as an example of how to gain insight into vulnerabilities (also see other approaches mapped, e.g., by Jeleff et al., 2022) and then structure engagement activities that address those vulnerabilities. Several of the researchers reiterated, though, that the resources available as well as their capacity for community mobilization did not allow them to pursue a substantial program of advocacy or policy work. The activities described here can therefore not be considered comprehensive CE on their own but rather as one step of a long-term process—for which adequate funding and structural support (by, e.g., government and funding agencies) are needed. This question is, however, a fundamental part of the greater discussion within the practice of CE and reflects the need for sustainability and institutional integration in working with communities (Reynolds & Sariola, 2018). Further, the common challenges described here reinforce the need for researchers to remain highly adaptive to the local context in which engagement takes place. The study describes several approaches to ensure representatives of potentially more vulnerable groups in the CE process; nevertheless, whether this effectively ensures their input in the uptake of actionable solutions remains uncertain. The diverse social and political circumstances that research teams navigated do not lend themselves to a single set of steps or “lessons learned”; future researchers may instead apply the themes described above to other CE activities.

### Implications for the Field

Some key lessons are also seen in the applicability of the conceptual framework implemented here (Figure 1) to the broader field of community-based responses to infectious diseases and other crises. It was seen in each of the study sites described here that context and relationality are central to the operation of all aspects of CE. We see this in the forms of vulnerability that emerged through VA and were used as starting points for engaging with certain groups. Here, we closely relate to Zarowsky et al.’s (2013) or Luna’s (2009) conceptualizations of vulnerability as free from *a priori* assumptions or characterizations of groups, and instead leading with systematic qualitative data collection. Research groups then took on a more critical view of vulnerability, as Domingues (2022) describes, calling into question the tension between COVID-19 state interventions targeting individual behaviors, and the existing, disenfranchising contexts in which communities were already found. This kind of analysis, where insights on vulnerabilities contributes to CE efforts, is a natural entry point for social science researchers, especially in identifying how engagement practices and larger interventions are entwined with local bottom-up practices.

We also see the dynamics of relationality in the engagement process itself, where researchers found themselves in positions of knowledge brokers, project managers, or policy makers. Descriptions of the role of the researcher in more action-oriented research have been described in the past. Indeed, Rosendahl et al. (2015) advocate for a reflexivity with a full recognition of the power relationships between the researcher and the researched, the researcher and their institutions, and all of the dynamics that fall in between. This is reflective of the framework used for this study (Osborne, Paget, Giles-Vernick et al., 2021), as well as other recent reflections of CE (MacGregor et al., 2022; Popay et al., 2021) and long-standing theoretical discussions on the role of variously situated actors and their claims to knowledge (Latour & Woolgar, 1979). These important insights are derived from the reflective exercises carried out by the project teams; but as the impact of the engagement activities described in this paper is not possible to describe here, we similarly do not seek to prescribe a set of steps for implementing CE beyond the guiding principles and entry points for social science research, detailed above.

## Conclusion

In this project, we developed and piloted an adaptable CE approach that focuses on addressing mechanisms of vulnerability to the spread and consequences of COVID-19. We described the experiences in piloting this action framework in relation to vulnerability in the context of infectious diseases, in which qualitative data provide an evidence base for action-oriented dialogue among community stakeholders and researchers.

The method may be used in research and implementation of recommendations regarding vulnerabilities elsewhere, in the context of infectious diseases and beyond. Likewise, the lessons drawn from doing CE in this project in five European countries are beneficial for applying social and behavioral science methods to inform public and community engagement, as well as contribute to policy recommendations.

Insights from working with these different CE case studies show that forming the engagement process based on qualitative data and flexible understandings of community are vital. Working closely with local stakeholders was key to forming appropriate policy recommendations that addressed vulnerabilities discovered through qualitative insights. The actual value for vulnerability reduction in practice depends on implementation success, effectiveness, and involvement of different community stakeholders.

Implementation, uptake, and integration of recommendations is a complicated and often longitudinal process, and challenges and obstacles will vary per community, the stakeholders involved, and the political and social landscape. We hope that the insights on CE to address mechanisms underlying vulnerabilities engendered by the Sonar-Global project will contribute to a future in which communities can take ownership to address the (health) issues that affect their lives and fully integrate their recommendations into institutionalized approaches.
